# ERRATUM: Performance of *Trauma and Injury Severity Score* (TRISS) adjustments: an integrative review

**DOI:** 10.1590/1980-220X-REEUSP-2024-ER19en

**Published:** 2024-09-30

In the article “Performance of *Trauma and Injury Severity Score* (TRISS) adjustments: an integrative review”, with the DOI: https://doi.org/10.1590/S0080-623420150000700020, published by the journal: “Revista da Escola de Enfermagem da USP, volume 49 (spe) of 2015, p. 135–143, on page 143:

**Now read:**

Include the section:

## Financial support

This study was financed in part by the Coordenação de Aperfeiçoamento de Pessoa de Nível Superior – Brasil (CAPES) – Finance Code 001.

