# Supplementary material for: ERRATUM: Performance of Trauma and Injury Severity Score (TRISS) adjustments: an integrative review
Source: Rev Esc Enferm USP. 2024 Sep 30;58:e2024ER19. doi: 10.1590/1980-220X-REEUSP-2024-ER19en (PMC11468611; doi:10.1590/1980-220X-REEUSP-2024-ER19en)
Supplement: Translation

# Translation: ERRATA: Desempenho dos ajustes do *Trauma and Injury Severity Score* (TRISS): revisão integrativa

No artigo “Desempenho dos ajustes do *Trauma and Injury Severity Score* (TRISS): revisão integrativa”, com número de: DOI: https://doi.org/10.1590/S0080-623420150000700020, publicado na Revista da Escola de Enfermagem da USP, volume 49 (spe) de 2015, p. 138–146, na página 146:

**Leia-se:**

Incluir a seção:

## Apoio financeiro

O presente trabalho foi realizado com apoio da Coordenação e Aperfeiçoamento de Pessoal de Nível Superior – Brasil (CAPES) – Código de Financiamento 001.
